# Complete Genome Sequences of Penicillin-Resistant Bacillus anthracis Strain PCr, Isolated from Bone Powder

**DOI:** 10.1128/MRA.00670-19

**Published:** 2019-08-29

**Authors:** Akiko Okutani, Satoshi Inoue, Shigeru Morikawa

**Affiliations:** a Department of Veterinary Science, National Institute of Infectious Diseases, Tokyo, Japan; University of Arizona

## Abstract

Bacillus anthracis, the etiologic agent of anthrax, is susceptible to beta-lactam antibiotics, but few cases of naturally occurring penicillin-resistant strains have been reported. We report the genome sequence of penicillin-resistant strain Bacillus anthracis PCr, isolated from imported bone powder in 1978 in Japan.

## ANNOUNCEMENT

Anthrax caused by Bacillus anthracis, a spore-forming Gram-positive bacterium, is one of the most severe zoonoses and poses serious threats to both public and animal health. Bacillus anthracis is susceptible to beta-lactam antibiotics, and penicillin is the antibiotic recommended for postexposure prophylaxis and for treatment of anthrax cases (1). Chen et al. (2) reported that 2% to 16% of isolates were resistant to beta-lactams, and it is known that there are several mutation types within the penicillin-resistant B. anthracis genome.

We discovered a penicillin-resistant B. anthracis strain, PCr, stored as spore solutions in a freezer at the National Institute of Animal Health in Tsukuba, Ibaraki, Japan, while searching the past isolates of B. anthracis stored in Japan to construct national strain archives (3, 4). The strain PCr was originally isolated from imported bone powder from Thailand, which was fed to cattle diagnosed with anthrax in 1978 in Shizuoka Prefecture in Japan (5). In this study, we analyzed its whole genome using long-read sequencing technology. One loop of droplet of spore solution was cultured on an LB (6) agar plate. Then, a colony was inoculated to LB broth for DNA extraction using the phenol-chloroform method (7). A 20-kb library was prepared with the single-molecule real-time (SMRT) cell template prep kit and sequenced with 1 SMRT cell run of PacBio RS II P6-C4 technology. After mapping of single-pass reads to seed reads was conducted, a consensus sequence of the mapped reads was generated. Some reads that were fully contained in other reads or that had too high or too low overlaps were filtered out to obtain a consensus sequence with higher quality. The high-quality filtered 155,952 subreads with an average read length of 9,947 bp (*N*_50_ value, 14,209 bp) were assembled using the Hierarchical Genome Assembly Process v3 (RS_HGAP3), with default parameters (8). Three contigs were assembled, and when the two ends of a contig overlapped, the contig was connected into a circular form. Error correction and polishing were performed with Quiver (8). Annotation was performed using the rapid prokaryotic genome annotation tool Prokka v1.13 (9). The total length of PCr was 5,209,717 bp, with 5,467 putative coding sequences (CDS) and an average GC content of 35.4% for the chromosome; the total length of plasmid pXO1 was 181,773 bp, with 203 CDS and a GC content of 32.5%, and the total length of plasmid pXO2 was 94,736 bp, with 108 CDS and a GC content of 33.0%.

**FIG 1:**
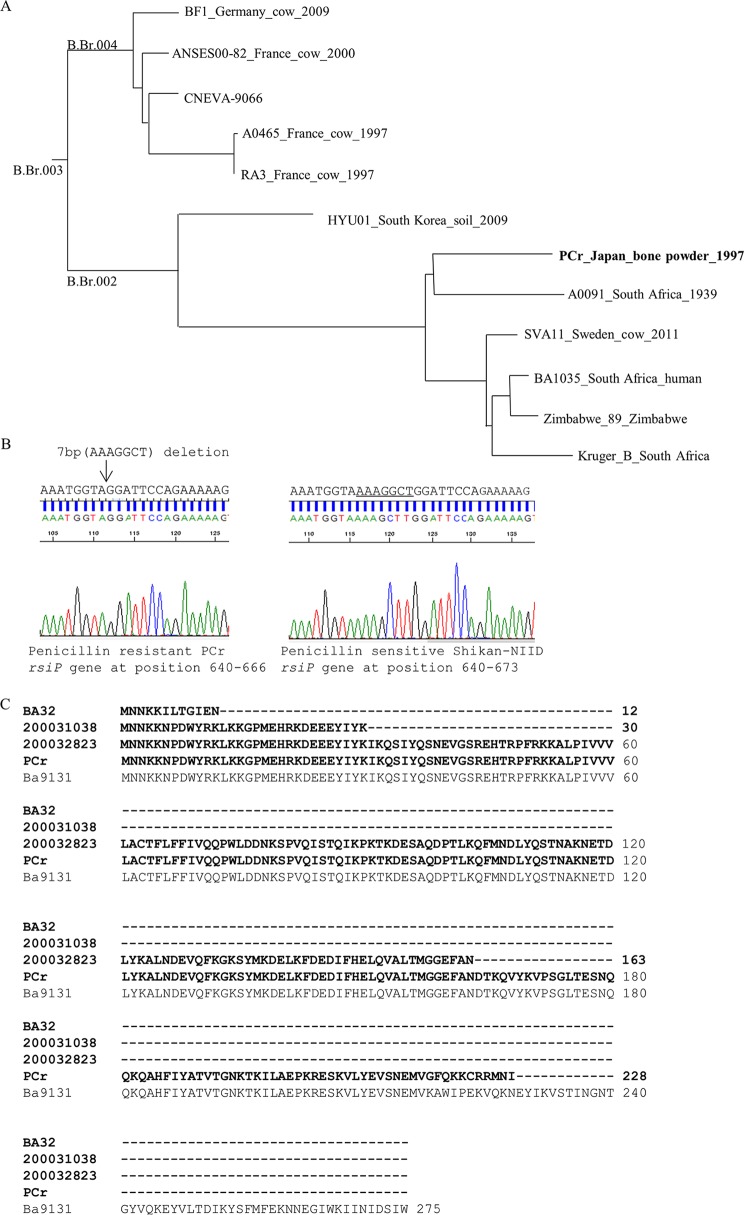
(A) A total of 2,177 core-genome SNPs (11) were analyzed in 12 B. anthracis strains belonging to the B.Br.001/002 lineage, including the penicillin-resistant PCr. The evolutionary tree was inferred using the maximum parsimony method conducted in Molecular Evolutionary Genetics Analysis (MEGA X) (16). The consistency index was 0.996708. The names of the published diagnostic SNPs (10) are labeled, and strain PCr is indicated by bold text. (B) Sequence electropherogram of the PCR product of the *rsiP* gene of penicillin-resistant strain PCr showing a 7-base deletion at position 652 and at the same region of the penicillin-sensitive strain Shikan-NIID. (C) Alignment of the amino acid sequence of RsiP of the penicillin-resistant B. anthracis strains BA32 (13), 200031038 (14), 2000032823 (14), and PCr and penicillin-susceptible B. anthracis Ba9131 (13). The amino acid sequences of penicillin-resistant strains are indicated by bold text.

The phylogenetic analysis of strain PCr using *in silico* canonical single-nucleotide polymorphism (canSNP) typing (10) and core-genome SNP analysis with an analytical pipeline (11) were performed as previously described (4). These analyses revealed that PCr belonged to the B.Br.001/002 lineage, which mainly contained strains of African origin (12) (Fig. 1A). Strain PCr possessed a novel frameshift mutation in the β-lactamase regulator gene *rsiP*, producing a 7-base deletion at positions 652 to 658 (Fig. 1B), which was predicted to result in a truncation of 228 amino acids (aa) in the RsiP protein (Fig. 1C). Several types of mutations in *sigP* and *rsiP*, an extracytoplasmic function sigma factor and its cognate anti-sigma factor gene, have been reported in the genomes of penicillin-resistant B. anthracis strains (13–15). The mutation in the PCr strain is likely to be related to its penicillin resistance, and further investigations are warranted.

### Data availability.

The sequences have been deposited in DDBJ/ENA/NCBI under the accession numbers AP019731 (chromosome), AP019732 (pXO1), and AP019733 (pXO2) for PCr. The raw sequence reads were deposited in the DDBJ Sequence Read Archive (DRA)/NCBI SRA under accession number DRA008501.
